# Keystroke dynamics in the pre-touchscreen era

**DOI:** 10.3389/fnhum.2013.00835

**Published:** 2013-12-19

**Authors:** Nasir Ahmad, Andrea Szymkowiak, Paul A. Campbell

**Affiliations:** ^1^CICaSS Group (Concepts & Innovation in Cavitation and Sonoptic Sciences), Carnegie Physics Laboratory, University of DundeeDundee, UK; ^2^Division of Molecular Medicine, College of Life Sciences, University of DundeeDundee, UK; ^3^School of Science, Engineering and Technology, University of Abertay DundeeDundee, UK

**Keywords:** keystroke analysis, pre-touchscreen, security, authentication, identity

## Abstract

Biometric authentication seeks to measure an individual’s unique physiological attributes for the purpose of identity verification. Conventionally, this task has been realized via analyses of fingerprints or signature iris patterns. However, whilst such methods effectively offer a superior security protocol compared with password-based approaches for example, their substantial infrastructure costs, and intrusive nature, make them undesirable and indeed impractical for many scenarios. An alternative approach seeks to develop similarly robust screening protocols through analysis of typing patterns, formally known as keystroke dynamics. Here, keystroke analysis methodologies can utilize multiple variables, and a range of mathematical techniques, in order to extract individuals’ typing signatures. Such variables may include measurement of the period between key presses, and/or releases, or even key-strike pressures. Statistical methods, neural networks, and fuzzy logic have often formed the basis for quantitative analysis on the data gathered, typically from conventional computer keyboards. Extension to more recent technologies such as numerical keypads and touch-screen devices is in its infancy, but obviously important as such devices grow in popularity. Here, we review the state of knowledge pertaining to authentication via conventional keyboards with a view toward indicating how this platform of knowledge can be exploited and extended into the newly emergent type-based technological contexts.

## OVERVIEW: AUTHENTICATION

With the magnitude of online and computer-based systems and services increasing rapidly over recent decades, the need for enhanced computer security has become a significant concern. Accurate authentication of user identity is of paramount importance, and the following techniques are most often used toward that objective (Wood, 1977):

• A unique (“hidden”) electronic key is employed, known only by the user, most commonly a password or PIN (personal identification number), which serves to access the system.
• A physical security measure (e.g., formal identification/swipe card) is used to identify the user. Systems often use such objects in conjunction with a hidden key.
• A biometric authentication system may be used such that a user’s unique physical or behavioral traits are inspected for verification of his/her identity by comparison with a validated database record.

Currently, systems most commonly in use prompt for a hidden password alongside an identifying username. These systems often recommend that the password used should be a completely unique, complex, and long entry that is not used for any other purpose. In reality, most users find remembering different sets of long alpha-numeric sequences for each and every service impractical, and tend to reuse the same password for more than one service. Alternatively, users might record their passwords, either electronically or on paper. Moreover, in order to assist password recall, users will often create a password or PIN which is in some way related to a personal aspect of their lives (e.g., birthdays and names). Recording and repetition of passwords obviously compromises the hidden requirement for their unique key’s security, opening the way for intruder access. Furthermore, passwords which are based upon the personal details of a user’s life can be susceptible to dictionary or “brute force” hacks, as well as educated guesses made by an informed imposter.

Systems with physical security measures also represent security issues, as the physical nature of the tokens/keys makes them prone to theft, or the data within them may be simply cloned, again compromising the target service. The implementation of further security layers is therefore a critical goal of biometric authentication.

Biometric authentication and identification are methods whereby unique physiological attributes or characteristic traits of individuals are used to verify their identity. Analyses of an individual’s fingerprint or unique iris pattern are two of the most widely used security techniques in this field. Although these methods are a great deal more secure than a single password, their significant setup costs and the intrusive nature of scanning makes them impractical for many purposes. Regardless of cost, it must be recognized that such systems remain fallible, but at the moment still prevail as the most accurate route to authentication available.

Analysis of keystroke dynamics is an alternative approach to biometrics authentication. This technique makes use of the natural pattern and manner in which a user types at a keyboard to verify their identity. In moving toward the establishment of a validated record, a user must initially be enrolled within a system, whereupon the user’s typing pattern is recorded and stored within the system. This record can then be consulted/compared when the user attempts to gain access to his/her system. This type of authentication would be implemented within a login system such that a user’s entry of their username and password is analyzed – thereby adding a new layer of security to the existing systems.

### A BRIEF HISTORY OF KEYSTROKE DYNAMICS

Of the early documented research and analysis into keystroke dynamics authentication, the insightful and thorough article by Gaines et al. (1980) is particularly illuminating. Their research showed that the field was effectively initiated during the initial manual phase of telegraphy, where operators had been observed to have a unique “fist” (tapping style) by which their colleagues could often identify them. By extrapolation of that principle, they hypothesized that a similar signature could arise during regular typing and a preliminary analysis was conducted, investigating the relevance and effectiveness of a system of identification of individuals, based upon their unique keystroke signatures. While Gaines et al. (1980) concluded that such a system *could* be effective as a tool for authentication, they acknowledged that the findings were only based on a small sample, using the data from seven touch typists, their task having been to type three distinct sections of text, some 4 months apart. Moreover, not every typist was available for each repeat session. Despite this small number of subjects, the researchers were able to observe and differentiate between their differing typing styles.

The study by Gaines et al. (1980) popularized the use of digraph data, i.e., data associated with two successively typed letters (viz. *in*, *io*, *no*, *on*, and *ul*) – a method that paved the way for many subsequent keystroke analysis groups to forge a first path into the field and which has remained popular with analysts. Following this preliminary assessment of the viability of keystroke analysis, other researchers pursued different routes for user identification and authentication, with an emphasis on the reduction of two error rates, i.e., false acceptance rate (FAR) and false rejection rate (FRR). FAR involves the mistaken acceptance of imposters, i.e., false positives; FRR is the error associated with the false rejection of valid users, i.e., false negatives. By altering the threshold for acceptances (or rejections), FAR and FRR can be optimized to generate a measure of equal error rate (EER), that is, when FAR is equal to FRR. The use of this measure allows for a comparison of the accuracies across studies that may use different authentication methods and subject numbers.

Subsequently, research arising in the 1980s and 1990s (e.g., Umphress and Williams, 1985; Young and Hammon, 1989; Bleha et al., 1990; Joyce and Gupta, 1990; Obaidat and Macchairolo, 1993, 1994; De Ru and Eloff, 1997; Lin, 1997; Monrose and Rubin, 1997; Obaidat and Sadoun, 1997, 1999; Robinson et al., 1998; Coltell et al., 1999; Monrose et al., 1999; Tapiador and Sigüenza, 1999) began to explore alternative methods of keystroke analysis, typically employing a range of novel mathematical analysis techniques, but also differing in the formal data collection method. Statistical techniques (Gaines et al., 1980; Umphress and Williams, 1985; Young and Hammon, 1989; Bleha et al., 1990; Joyce and Gupta, 1990; Bleha and Obaidat, 1991; Monrose and Rubin, 1997; Robinson et al., 1998; Coltell et al., 1999; Monrose et al., 1999; Obaidat and Sadoun, 1999), neural networks (Obaidat and Macchairolo, 1993, 1994; Lin, 1997; Obaidat and Sadoun, 1997), and fuzzy logic (De Ru and Eloff, 1997; Tapiador and Sigüenza, 1999) have all been used in attempts to increase the accuracy and effectiveness of keystroke authentication. The data collected for use with these techniques were not only recorded directly by the computer being actively used, but also collected via a local network or server arrangement (Bleha et al., 1990; Bleha and Obaidat, 1991; Obaidat and Macchairolo, 1993; Tapiador and Sigüenza, 1999), showing that such keystroke authentication could be implemented in an online system.

A further innovation at this stage was that the keystroke analysis system could be implemented not only to authenticate users during login, but also to make that judgment more robust by recording/monitoring keystrokes during the downstream session – whilst they wrote documents/emails. If an intruder was detected, some action would be taken by the system to limit access. Formally, keystroke analysis completed only at log-in became known as *Static Analysis* while that undertaken during the entire user session is known as *Continuous Analysis*.

Research in the most immediate past (Changshui and Yanhua, 2000; Cho et al., 2000; Haider et al., 2000; Monrose and Rubin, 2000; Wong et al., 2001; Bergadano et al., 2002; D’Souza, 2002; Henderson et al., 2002; Mantyjarvi et al., 2002; Eltahir et al., 2003, 2004, 2008; Jansen, 2003; Nonaka and Kurihara, 2004; Peacock et al., 2004; Araújo et al., 2005; Chang, 2005; Lee and Cho, 2005; Rodrigues et al., 2005; Curtin et al., 2006; Hosseinzadeh et al., 2006; Lv and Wang, 2006; Clarke and Furnell, 2007; Hocquet et al., 2007; Loy et al., 2007; Grabham and White, 2008; Lv et al., 2008; Saevanee and Bhatarakosol, 2008, 2009; Campisi et al., 2009; Hwang et al., 2009a,b; Killourhy and Maxion, 2009; Revett, 2009; Nguyen et al., 2010; Chang et al., 2011, 2012; Giot et al., 2011; Karnan et al., 2011; Teh et al., 2011; Xi et al., 2011) has incorporated newly developed mathematical and data recording techniques – again employing statistical techniques and neural networks, but also attempting to fuse data from multiple parallel sensors. The types and differences between the various mathematical techniques are discussed in the next section. Other than new analysis techniques, novel types of data were also considered and analyzed. For example, existing keyboards were modified to generate a measure of the pressure with which a user presses a single key (Henderson et al., 2002; Eltahir et al., 2003, 2004; Nonaka and Kurihara, 2004; Lv and Wang, 2006; Hocquet et al., 2007; Loy et al., 2007) – the aim of which was, again, to increase the veracity of user analyzed identity. Such pressure measurements proved useful in building a more accurate template of users’ unique keystroke patterns. Keyboard modification was generally achieved by addition of an analog electronic component sensitive to pressure, or some indirect measure of pressure (e.g., piezo-resistive film) was either placed between the keyboard and the surface upon which it sat, or alternatively, beneath a number of active keys. On-board microphones (Nguyen et al., 2010) could also be employed to take an indirect measure of pressure, based upon the characteristic acoustic signature arising.

The increased demand for security in other areas of modern technology has also led to keystroke dynamics research having been carried out on mobile phones, e.g., button-based (Clarke and Furnell, 2007; Campisi et al., 2009; Hwang et al., 2009a) and touch-based devices (Mantyjarvi et al., 2002; Saevanee and Bhatarakosol, 2008, 2009); numerical keypad systems (Mantyjarvi et al., 2002; Grabham and White, 2008), and also web-based systems (Bleha et al., 1990; Bleha and Obaidat, 1991; Obaidat and Macchairolo, 1993; Tapiador and Sigüenza, 1999; Cho et al., 2000; Curtin et al., 2006). The applications of these systems are discussed alongside a measure of the accuracy of each method in subsequent sections.

It should be noted that research undertaken in this field tends to make use of different sets of data: studies generally have different numbers of subjects, and employ different sets of “test text” as authentication samplers. For example, some studies require the subjects to type out a username and password combination (of relatively short length) whilst other studies request the input of a large section of text. The difference in methodologies provides a challenge for making direct comparisons among papers using the stated error rates alone. Furthermore, the papers described below make use of different classes of keystroke latency. In principle, four types can be used: the timing for a key to go “Down–Up” (hold time), “Down–Down,” “Up–Down,” and “Up–Up.” Different combinations of these four latencies have been exploited by different groups and a specific choice may affect the indicative error rates arising.

## KEYSTROKE DYNAMICS FOR SECURITY

### MATHEMATICAL ANALYSIS TECHNIQUES

The mathematical approaches to keystroke analysis can be divided into the following groups, all of which are discussed below:

• Statistical techniques
• Artificial neural networks
• Fuzzy logic
• Other

#### Statistical techniques

Statistical analysis of keystroke dynamics is perhaps the most researched avenue within the field. Initially, basic statistical features such as the mean and standard deviation of keystroke timings were utilized, however, these were quickly expanded upon to ascertain the detection of anomalies and irregularities of timings.

*t*-test analyses were prevalent in the earliest reports. This method of analysis required the mean values of two samples to be taken and compared, in order to determine whether the two samples emanated from the same original source (typist). In the case of keystroke dynamics, the *t*-test analysis was used not only to compare the mean, but also the standard deviation of inter-key latencies (Gaines et al., 1980; Umphress and Williams, 1985).

In the work by Gaines et al. (1980), a group of repeated digraphs was analysed using this method, and with subjects typing comparatively large amounts of text, this technique proved effective. It should be noted, however, that with regular password strings, the digraphs are not repeated sufficiently often for this technique, in the form alluded to, to be appropriate for a login-based analysis of keystroke dynamics. However, although this technique may not be directly applicable to [short] login keystroke analysis, the accuracy rates, as mentioned above, proved encouraging, and certainly provided the initial indications that statistical analyses could be sufficiently accurate for authentication in the context of computer systems.

Nowadays, techniques exploiting the features of statistical analysis often combine the mean and standard deviations for keystroke latencies as reference data (Joyce and Gupta, 1990; Robinson et al., 1998; Araújo et al., 2005). The data are collected when users are initially registered into a system, whereupon it is required to enter their authentication string (e.g., password) multiple times. The latency times are combined to create a “vector.”

Many reports have used a variation on this technique, combining it with an intrinsic threshold so that when a user attempts to access the system, the latencies of the entered authentication string are compared against the reference signature. If the differences between the two are within the threshold, the user is accepted. For example, Araújo et al. (2005) used four keystroke features, each with 10 character long password strings. Using the mean and standard deviation, a template for each keystroke feature for each element was made and stored. Interestingly, this approach was tested not only by valid users and imposters, but also “observer” imposters, such that these subjects were allowed to view the valid subject’s typing style. In the event, Araújo et al. (2005) were able to achieve an error rating of FRR = 1.45% and FAR = 1.89%, an impressively high outcome for this style of statistical analysis.

Other studies have also made use of Bayesian analysis (Bleha et al., 1990; Bleha and Obaidat, 1991) in an attempt to achieve a lower rate of misclassification. This technique treats the pattern vector as a multivariate probability density function, and the analysis, when combined with a minimum distance classifier, was used extensively in attempts to gain accuracy. Minimum distance classifiers define the difference between two samples as an index of similarity. This can be beneficial in keystroke dynamics in that setting a threshold for this minimum distance allows a user to be authenticated in a keystroke analysis system within a threshold unique *to their own* variation in keystroke signature.

Other statistical analysis techniques include methods of distance classification and probability measures (weighted and non-weighted; Monrose and Rubin, 1997; Robinson et al., 1998). Auto-regressive (AR) and AR moving-average (ARMA) models were considered with and without measures of pressure (Changshui and Yanhua, 2000; Eltahir et al., 2004). Hidden Markov models (HMMs) have been implemented (Chang, 2005) with a similarity histogram, and, by attempting to recognize patterns, produce promising results. Gaussian mixture models (GMMs) have also been tested and found to attain low (under 3%) error rates (Hosseinzadeh et al., 2006). Moreover, combined multiple techniques have had their distinctive advantages, such as the fusion of a statistical method, a measure of disorder between feature vectors and time discretization (Hocquet et al., 2007). Teh et al. (2011) completed a multi-layer fusion of a Gaussian probability density function (GPD) and a directional similarity measure (DSM) attaining an EER of circa 1% with a “Multiple Layer Multiple Expert” fusion technique employing AND voting rules. This approach generally yields better error rates than many other fused analytical procedures, for instance, those making use of statistical and fuzzy logic approaches.

#### Artificial neural networks

Artificial neural networks (ANNs) are mathematical (or computational) models that imitate, and are inspired by, the function and processes in a biological neural network. The system is built using artificial neurons with well defined connection prescriptions. These ANNs can be utilized to extract complex connections and patterns in data.

In the context of keystroke dynamics, the input to the ANN is largely the timing between successive keystrokes. These keystroke timings are then computed through the network comparator to pre-collected and validated data, in order to determine whether the user is authentic. “Back-propagation” neural networks are usually implemented, which are feed-forward networks employing multiple layers between the input and output nodes.

The initial use of ANNs was to aid in user identification using keystroke dynamics (Obaidat and Macchairolo, 1993, 1994) and several of the first wave of studies to implement such a neural network approach simply used users’ keystroke latencies as the basis for discrimination. It was found that a hybrid “sum-of-products” network gave the least error: this type of network consists of a simple back-propagation setup between the input and hidden layers followed by a sum-of-products connection between the hidden and output layers. This sum-of-products technique acts in such a way that the output of one node is the weighted sum of the inputs from multiple other nodes. The majority of the ANN systems use some variation on this technique, although in many cases, the ultimate analysis is completed by different types and complexities of the system. ANNs deliver reasonably high accuracy, 97.8% Obaidat and Macchairolo (1993) and 96.2% for the same technique in Obaidat and Macchairolo (1994) with a short neural network training time (~1 min training). However, in this case it is important to note that the system was typically used for identification only, i.e., the user keystrokes were matched against a database to find the closest match. Thus, accuracy was not an indication of how well the system was able to identify imposters.

Several research groups subsequently began to investigate the application of ANNs in verification, as a competing technique to statistical analysis. Here, one of the most successful research studies into this area was undertaken by Obaidat and Sadoun (1997), who tested both statistical and neural network approaches to keystroke dynamics verification and achieved zero percent error rates (EER) for learning vector quantization (LVQ), radial basis function networks (RBFN), and Fuzzy ARTMAP neural networks (i.e., a neural network architecture based on the synthesis of fuzzy logic and adaptive resonance theory). Whilst this result was extremely promising, it should be noted that the extent of data sampling required on participants was considerable and therefore poses limits on the implementation of such systems. Over the course of an 8-week experiment, 15 “valid” users provided 225 sequences, and 15 “invalid” users provided 15 samples each. The samples taken from invalid users were used to “train” the system, whereas in a realistic system, there would be no access to invalid user keystrokes for such training purposes (unless it would be an integral part of an intense enrolment procedure). Nevertheless, the strength of such studies is that they underscore the applicability and potential for neural network approaches as part of the authentication/verification strategy. These same authors also discuss, and conclude, that the duration over which keys are held (hold time) is a better measure for keystroke signature than the time between key presses (inter-key time). However, the combination of *both* these timing sets serves to reduce errors.

Around the same period, Lin (1997) made first use of a dynamic multi-layered back-propagation neural network. This approach operated with distinct weightings being assigned to the keystroke latencies as they progressed through the system. These weightings were based on training sample data, and were constructed such that the root mean square error was reduced to an appropriate threshold. This study was able to validate users with a very low error – with FAR reaching lows of 0% and FRR = 1.1%. Although the error ratings were somewhat higher than those by Obaidat and Sadoun (1997), a much larger number of participants was tested (90 valid users and 61 invalid users) and intruder samples were not trained within the system, lending feasibility to its implementation.

More recently, Cho et al. (2000) developed a web-based neural network identity verification system and were able to attain very low error rates (average FRR error of 1% when FAR was 0%) using a multi-layer perceptron (MLP). Here, 25 valid users supplied 150–400 samples, with the last 75 being selected for testing. In parallel, 15 invalid users supplied five imposter attempts for each user, again resulting in 75 test signatures. The system was not required to be trained with the imposter signatures, however, the number of training signatures supplied by the user (75–325) would likely be too large, unless a continuous analysis were practical in the context of the application. A web-based system was also implemented using a Java applet that could be run within a web browser to connect to the server, illustrating that the system is available for electronic commerce applications.

The final notable approach within this category, k-NN, or k-nearest neighbor algorithms, has also been used with neural networks in order to accomplish pattern recognition. Wong et al. (2001) used a Euclidean distance measure for the nearest neighbor classification, however, the error rates achieved in this case were generally worse than those from the other studies employing neural networks.

#### Fuzzy logic

Fuzzy logic is a type of probabilistic logic that deals with reasoning that is approximate rather than fixed. For example, where other “crisp” logic systems have only two states (true/false, on/off) fuzzy logic makes use of the multi-valued interval between these states.

De Ru and Eloff (1997) made use of fuzzy logic as an analysis technique for keystroke dynamics. Here, the group used not only the time intervals between successive characters but also a measure of the typing *difficulty* of successive letters. This classification of difficulty was based upon the distance of the keys involved, and whether or not any of them were capitalized or had a range of whole number values. The time interval between two successive keystrokes was also identified using fuzzy logic, and subsequently binned within subsets: very short; short; moderately short; and somewhat short. By combining the timing and typing difficulty, a specific keystroke combination was assigned to categories within some degree (e.g., 20% high and 40% short etc.). Using all of these variable and approximately 20 “fuzzy” rules, the group created a system of keystroke analysis which was able to function, but with some error.

Tapiador and Sigüenza (1999) created an Internet-based keystroke analysis system that made use of a username and password to create a fuzzy template. When a user then attempted to log-in, the sample was compared to the fuzzy template for authentication. Whilst the use of simple username and passwords aided the accuracy of their keystroke analysis system, it could, however, lead to intruders being more readily able to ascertain this access password. The authors did not provide any detailed information on error rates, however, and the statistically small sampling with only nine participants might limit the generalizability of this study.

#### Other

Although the majority of research in this field focussed on the application of digraph and inter-key latencies, some studies also approached the field with other techniques such as trigraph latency. Bergadano et al. (2002) made use of such trigraph latencies in a novel approach to keystroke dynamics analysis. They achieved a reasonably competitive error rate (4% FRR and 0.01% FAR). The use of 154 participants is statistically favorable compared with many other studies in this field, however, participants were tasked to enter a text consisting of 683 characters, which could be perceived as cumbersome or impractical for covert implementation. In this study, the data analysis was unique in that the group used mathematical techniques to arrange trigraphs in order of increasing typing time for each word. This created a “model” for that user such that when users subsequently attempted to access the system, their typing sample was compared to their specific model: if the distance between the two was sufficiently small, the user was accepted.

Many studies have made use of large sections of text when attempting to verify a user’s identity. In some cases, this was simply to ensure that there were sufficient data to facilitate reliable keystroke analysis, however, as already highlighted, such systems would be less useful for applications for user verification with log-in strings (username and password). However, they do underscore the applicability and accuracy of a system which monitors free text in a continuous mode where a user’s typing style throughout their active session is assessed. Curtin et al. (2006) studied the feasibility of a system monitoring large sections of text by extracting information such as the means and standard deviations of typing times for the eight most frequent letters in the alphabet (e, a, r, i, o, t, n, s), the means and standard deviations of the transition times between the most common letter pairs (in, th, ti, on, an, he, al, er, etc.), variables related to the number of presses of special keys (delete, enter, shift, arrow keys, etc.), the number of times the mouse keys were used (also double clicks), and the total time duration of the text input. A nearest neighbor classifier using Euclidean distance was then used to compare test data to training data for identification purposes. The classifier achieved accuracies greater than 90% for recognition, with accuracies up to 100% under certain conditions (large sections of text and small participant size). This study showed the feasibility of this system, however, and importantly, did *not* test the system with imposter keystrokes to test detection in that context. Thus, this system could only be implemented to ensure that valid individuals were not making use of unauthorized machines, systems, or files.

Lee and Cho (2005) created a new system for classical keystroke dynamics that made use of valid and imposter training samples. Imposter samples become useful over time by the collection of data when imposters attempt to access a system, thus allowing for tightening of the signature of a user, so that the algorithm can more accurately identify valid and invalid users. After testing this system with six different analysis techniques, the one-class LVQ (1-LVQ) and support vector data description (SVDD) were found to be the most accurate, when inclusion of imposter samples were available. Although the inclusion of imposter samples in this case and others results in an increase in accuracy of the system, acquisition of such samples can be difficult. An imposter would first have to access the system knowing the password and be caught and identified as not being a valid user, whereas, if a valid user’s attempt was flagged as an imposter, the accuracy with which the valid user could be identified then might be reduced. Therefore, there remain significant issues with such systems at present.

### VARIABLES AND EQUIPMENT

#### Pressure

After attaining fairly high accuracies with keystroke latency analysis, investigations into other variables which could be used to aid this accuracy were developed. The most applicable and investigated addition was that of keystroke pressure. Measures of pressure were achieved by making use of piezo-electric and piezo-resistive sensors interfaced with the computer system to which the active keyboard was connected (Eltahir et al., 2003, 2004; Nonaka and Kurihara, 2004). These sensors were either placed beneath specific (or all) keys (Eltahir et al., 2003, 2004) or upon the support sections of the keyboard (Nonaka and Kurihara, 2004).

For verification, details of the key-specific pressure waveform, or its associated temporal characteristics, were stored, and were then consulted when a user attempts log-in. The use of this additional pressure variable was seen to increase the accuracy with which the users were validated, albeit with varying degrees of success.

Nonaka and Kurihara (2004) made use of pressure waveforms by placing two pressure sensing strips as the keyboard support beneath the “W” and “O” keys. In this case they not only used the waveforms to attain pressure measures but also as a means to more accurately measure keystroke timings. To attain these accurate measures of keystroke timing, they reduced the pressure waveform to a set of transforms equivalent to maximal overlap discrete Haar wavelet transforms (MOHWT). The system was used with a small number of subjects, however, details of testing were not provided.

Eltahir et al. (2004) implemented an AR classifier for use with creating pressure templates for user validation. Eltahir et al. (2008) developed this method further and used an AR classifier with stochastic signal modeling for the analysis of the pressure aspect of the keystroke signature. This pressure template was used to verify user identities and was integrated into a program called pressure-based biometric authentication system (PBAS). The system was created with a normal keyboard with embedded force sensors connected to a data acquisition system (filtering and amplification followed by a connection to a digital to analog PCI card in a PC). A measure of the Total Square Error (TSE) was used to discriminate between valid and invalid users. Here, the experiments were carried out with 23 participants and the group was able to achieve an EER of just over 3%.

Lv and Wang (2006) made use of pressure measurements for keystroke verification using three analysis methods. The three analysis methods consisted of a measure of global statistical features of the pressure wave (mean, standard deviation, difference between max and min, positive and negative energy centers), dynamic time warping of the waveform and traditional statistical keystroke analysis. These analyses were carried out after pre-processing of the waveforms using noise removal and normalization. The best error rates were achieved when each of the analysis techniques were weighted and applied. This resulted in an error rate of 1.41% EER, which was lower compared to the error rate when measures of pressure were removed, i.e., 2.04%. Thus, it is clear that pressure does indeed increase the accuracy of the verification, however, this small (0.63%) increase in accuracy should be evaluated based on the cost of the additional components required for pressure measurement, which are not available on typical keyboards.

Loy et al. (2007) used the ARTMAP-FD (FD – familiarity discrimination) neural network as a competing neural network analysis technique. In this case pressure was used by applying piezo-resistive force sensors beneath the keyboard matrix. After baseline subtraction, a fast Fourier transform (FFT) was used to transform the pressure time signals into frequency domain signals. Again, with the use of pressure, a reduction of 3.16% in EER was observed, however, the overall error was significantly higher than many other neural network and pressure-based applications (11.78% EER).

Other unique approaches that used pressure-based measurements were also implemented in systems such as by Nguyen et al. (2010). Here, a microphone was used to record the sounds produced by the keystrokes. The data from the microphone were then used to create a standardized “bio-matrix” detailing the keystroke timing and force, with data becoming extracted via an independent component analysis (ICA) routine. ICA extracted the data from the bio-matrix, and the Fast Artificial Neural Network library (FANN) was used for recognition and authentication. This technique proved to be competitive in terms of accuracy, achieving an FAR of 4.12% and an FRR of 5.55%. Furthermore, the use of a microphone represents a novel technique for acquiring pressure measurement, which could be much cheaper to implement than the alternative methods mentioned above. One obstacle to the use of microphones is that the results would be easily affected in the presence of noise – although it is fair to say that intelligent noise cancelation techniques are becoming main-stream even on civilian devices such as mobile phones.

**Table 1** serves to summarize, in terms of input demand, analysis methods employed, and respective accuracy rates, for several key examples from the various typing biometric approaches used thus far.

**Table 1:** Summary of salient typing demand, analysis mode, and accuracy rates for a spectrum of different keystroke biometric approaches.

Typing input demand	Method of analysis	Accuracy	Reference
10 character string input 10 times with 30 participants	Statistical ($\overset{-}{\chi }$ and ρ)	FAR = 1.89%	Araújo et al. (2005)
		FRR = 1.45%
Circa 40 character string input 10 times with 100 participants	Statistical (GPD fused with DSM)	EER ≈ 1%	Teh et al. (2011)
Circa 30 character string input 10 times with eight participants	Statistical (GMM)	FAR = 2.1%; FRR = 2.4%EER < 3%	Hosseinzadeh et al. (2006)
Short phrase entry with six participants	Artificial Neural Net	Accuracy = 97.8%	Obaidat and Macchairolo (1993)
15 valid and 15 invalid users × 225 sequence	ANN + Fuzzy logic	EER = 0%	Obaidat and Sadoun (1997)
Short password entered three times with 90 valid and 61 imposter participants	Multilayer back propagated ANN	FAR = 1.1%; FRR = 0%^*^	Lin (1997)
7 character string input between 150 and 400 times with 25 participants	ANN using multilayer perceptron	FAR = 0%; FRR = 1%	Cho et al. (2000)
At least 8 character string input 25 times with 29 participants to study	Fuzzy logic	FAR = 2.79%; FRR = 7.379%	De Ru and Eloff (1997)
683 character string using 154 participants	Statistical - trigraph-based	FRR = 4%; FAR = 0.01%	Bergadano et al. (2002)
Short (*n* < 15 characters) strings input 10 times with 23 participants	Auto-regressive classifier linked to pressure data	EER ≈ 3%	Eltahir et al. (2008)
10 character password input to database enrolment with 50 samples (30 genuine and 20 forged)	Statistical; & Statistical augmented with pressure data	EER = 2.04%; EER = 1.41% (P-augmented)	Lv and Wang (2006)
8 character string with 10 timing- and 10 pressure vectors recorded	Artificial Neural Net augmented with pressure data	EER values of 16.5, 14.94, and 11.78% for respectively, pressure, latency, and pressure + latency	Loy et al. (2007)
Short string pairs input 15 times with 20 participants	Independent component analysis and fast-ANN augmented with acoustic record	FAR = 4.12%; FRR = 5.55%	Nguyen et al. (2010)

* With refined thresholding.

#### Handheld devices and mobile phones

With the large increase in the use, access, and ownership of mobile phones, the protection of personal and sensitive information within such devices is an obvious concern and authentication using keystroke dynamics could be a suitable addition to the current security measures. Such handheld devices have a number of limitations in terms of security (Jansen, 2003):

• Due to the small size, devices are easily stolen or misplaced.
• User authentication is by default disabled.
• Authentication systems on such devices can be very limited and easily deceived.

Keystroke dynamics analysis on such handheld and mobile devices could be somewhat more limited than that of a computer. It is also important to remember that most users do not type as often on mobile phones as they do on computers and so the detection of unique signatures could be more difficult. Moreover, the preferred typing style (with thumbs or one finger only) may not be directly correlated with standard keyboard operation.

Clarke and Furnell (2007) investigated the use of keystroke dynamics in the application of mobile phones. They made use of the numerical keypad on a large number of mobile phones before touch screens were introduced, and tested a number of neural network-based analysis techniques: feed-forward MLP (FF MLP); radial basis function (RBF); and generalized regression neural networks, finding the FF MLP network to be the most stable and useful in this case.

When acquiring samples for a numerical system, two sample sizes were used of four and eleven numbers. These string lengths were chosen as common PINs used to lock phones for security are often four numbers in length, and phone numbers themselves can be of lengths up to eleven numbers. Alphabetic input classification was conducted using samples from participants who were asked to type thirty text messages consisting of mixtures of quotes, lines from movies and typical text messages. In the case of typing letters on such first generation devices, keys had to be pressed multiple times to acquire the correct letters. Impressively, the study by Clarke and Furnell (2007) combined not only keystroke analysis but also voice, facial, and fingerprint recognitions, attaining very high accuracies. However, such systems require more mobile capabilities (camera or fingerprint reader) and a significant level of processing on the mobile phone.

In this context, Saevanee and Bhatarakosol (2008) used a k-NN approach with data (hold and inter-key times) from a numerical touchpad and were able to achieve accuracies of 99.9% with pressure measurements alone. A similar study (Saevanee and Bhatarakosol, 2009) used a probabilistic neural network (PNN) and achieved comparable results. The significance of the result is, however, once again tempered by the low subject numbers involved (only 10 participants with sample sizes of 10 characters measured at 20 ms intervals), while the stated accuracy using PNN (99%) is higher than that of others using different analyses. For example, Campisi et al. (2009) conducted keystroke dynamics analysis on mobile phones with telephone keypads, achieving an EER of 13%. A statistical analysis technique was implemented making use of four key hold and latency times for the typing of six 10-character passwords which were each repeated 20 times. The stated EER achieved was relatively high in comparison to implementation on a full keyboard which, using statistical techniques, typically report EERs of under 5% (see above).

Hwang et al. (2009a) applied keystroke dynamics analysis to four number PINs for mobile phones. Twenty-five participants took part and two different approaches were investigated, “Natural Rhythm without Cue” and “Artificial Rhythms with Cues.” The best results were achieved when the participants were required to use artificial rhythms – which reduced the EER to around 4%. A follow-up study by this same group into artificial rhythms (Hwang et al., 2009b) further elucidated the effects of pauses with cues, and attained sub 2% error rates. Chang et al. (2011) conducted a similar study investigating the feasibility of “click rhythm” based systems using mouse clicks, with EERs below 8%.

#### Keypads

Naturally, when considering the use of new security measures, keypad systems are important due to their current use in cash withdrawal systems or for controlling access to secure areas. Mantyjarvi et al. (2002) designed and made use of an unconventional keypad system. Their system implements an infrared receiver and transceiver system as a substitute for a button-based numerical input system. They then implemented an MLP and a k-NN algorithm to attempt keystroke verification. The accuracy achieved was affected by the implementation of this unique system, achieving classification results of 78–99% for k-NN, and 69–96% for MLP (the authors did not, however, provide details of the test data).

Using a similar setting, Rodrigues et al. (2005) used two analysis techniques to authenticate users using a numerical keypad, i.e., a statistical classifier and pattern recognition using a HMM. The statistical classifier exploited the means and standard deviations of keystroke timings and these were compared to any samples being tested by a measure of distance. The HMM produced the lowest error rate of 3.6% (EER) and although this is comparable to some error rates achieved by HMMs with full keyboards, the use of only the numerical keypad reduces the number of keys being pressed, making this finding relevant for implementation in actual keypad systems.

Grabham and White (2008) conducted similar tests, using the variables of applied force and key-press duration, which were coupled with a component-wise verification scheme and which resulted in a higher EER (~10%) when using an actual ATM keypad with individual force sensing devices beneath every key. Importantly, the keypad was designed to look and operate identically to an orthodox keypad system to ensure validity of the approach with a real-world scenario.

## NOVEL AND FUTURE APPLICATIONS

The field of keystroke dynamics has many other areas of use other than authentication. Lv et al. (2008) used pressure-based keystroke analysis for a completely novel application, where the pressure wave component was used as a technique for the detection of emotion. Fifty participants took part in their study, and were subjected to six different emotion inductions (neutral, anger, fear, happiness, sadness, and surprise) providing a total of 3000 samples and obtaining an accuracy of 93.4%. To induce the emotions, the subjects were asked to listen to and watch a short story for each emotion and immerse themselves in the situation when typing. Each individual emotional state was shown to produce a different pressure sequence. To analyze these different emotional states some initial pre-processing was needed (noise removal and normalization) and then three analysis techniques were fused together, including two pressure analysis approaches and one traditional keystroke approach. The two pressure analysis techniques included the analysis of Global Features of the pressure sequence and dynamic time warping as with Lv’s study (2006). The analysis was shown to be effective for these particular six emotions and as such, emotional state detection could have uses for many fields.

Lv et al. (2008) report that this emotional recognition system was used for intelligent game control and other applications. Feedback from a computer system based upon a user’s emotional state could be an interesting area of application, however, this research direction is still very much in its infancy. We suggest that the use of such an emotional recognition system could be relevant for controlling access to secure systems, in that emotional states such as anger or fear might be associated with critical states of the user that could potentially be monitored.

Other than the above analysis of emotional states for detection of different emotions, a similar analysis could also be applied for the detection of deception. Such a system could obtain a reference or baseline signature for a user and then, using keystroke data, attempt to identify when a user could be trying to deceive the system. For such categorization, a measure of the stress that the user is experiencing could be detected and analyzed. Investigation into such applications could use a greater number of variables than the typical keystroke analysis systems, as such measurements could increase the accuracy of the detection. Such analysis would most likely not be completed with an average keyboard, especially when pressure is a measure and so a more technologically advanced keyboard design is required.

Future keystroke analysis authentication tools could take to the Internet as web-based security systems for aiding in the security of online accounts and systems. For such systems to work effectively, they need to be able to complete keystroke analysis not only on traditional keyboards but also on touch-based devices. Investigation into the relationship between keystroke signatures obtained with traditional keyboards and those captured with touch-based systems could prove extremely useful. With the number of touch-based systems and tablet computers increasing rapidly in the last few years, such research could help to create a universal signature that could be used across platforms without need for multiple input data to each sensor. This research could lead to the development of such web-based keystroke analyses tools being a great deal more flexible in their use and ability.

## CONCLUSION

The application of keystroke dynamics to authentication has met with some compelling success, yet the standards continue to evolve in the drive toward optimal reliability. The accuracies achieved have reached heights of 99% with multiple techniques and with several data sets, proving that the use of such techniques would be valid and beneficial additions to current security systems. The analysis techniques used include statistical, neural network, and fuzzy logic approaches, and the inclusion of new parameter spaces such as pressure variables. The main variables against which the quality of the authentication systems have been measured are FAR, FRR, or EERs, which are ultimately the main indicators of the success of a biometric system. However, a comparison of different authentication methods based on these standard error rates is still challenging because of the heterogeneity of timing variables recorded (e.g., down–up, down–down, up–down, up–up times, digraphs, trigraphs, etc.). A comparison of different classifiers for user authentication appears to be only useful to the extent that they rely on the same variables.

Regarding the actual application of biometric systems, we conclude that ease of manner of enrolment should be a critical factor in determining the choice of a system, as this affects the practicality of the suggested biometric approach. For example, a number of the reviewed studies (Cho et al., 2000; Araújo et al., 2005; Lee and Cho, 2005) relied on imposter login attempts to refine the biometric system. The use of imposter data allows the specification of a more refined user profile and might be reasonable in the context of applications in which the user might expect to go through a specific enrolment procedure (e.g., access to secure military systems). However, relying on this approach is less practicable for systems that are used by standard, non-specialist users, as the ease with which individuals can be enrolled in a biometric authentication system becomes more relevant. A quick enrolment procedure using as few password and username characters would be preferable, however, few characters make the system more susceptible to classification errors. The balance of error rates and ease-of-use thus needs to be carefully determined, depending on the severity of the consequences of breaching a secure system.

Associated with this aspect is also the actual context of user enrolment. Enrolling via a server (e.g., Bleha et al., 1990; Obaidat and Macchairolo, 1993; Tapiador and Sigüenza, 1999), which could be an option for online banking, for example, shifts the responsibility of “proper” enrolment to the user. In a situation where the enrolment process is not controlled (e.g., accomplished in a structured environment and/or supervised by trained staff) the enrolment data might be “noisy,” thus increasing the likelihood of authentication errors. With emerging advances in authentication algorithms and technological developments, as well as sufficiently reliable systems, we would expect an increase in the actual implementation of such systems in the “real-world.” This also implies that the user-friendliness of such systems becomes more important for determining the success of the biometric application.

Other than the use of keystroke dynamics analysis with traditional keyboards, similar investigations have been carried out with other input devices such as touch screens and keypads. These used similar analysis techniques and were able to achieve accuracies close to those with full keyboards showing the applicability of this field to a range of devices and systems. Coinciding with an emerging interest in affective computing (Picard, 2000), keystroke analysis has also been implemented for other purposes, such as the detection of emotions. However, more research is needed in this avenue in order to achieve the maturity and reliability that traditional orthodox methodologies have achieved.

## Conflict of Interest Statement

The authors declare that the research was conducted in the absence of any commercial or financial relationships that could be construed as a potential conflict of interest.

## AUTHOR CONTRIBUTIONS

Nasir Ahmad conducted the associated lab-work that informed this paper, and wrote the draft, both under guidance from Paul A. Campbell. Andrea Szymkowiak and Paul A. Campbell corrected and updated the manuscript.
